# Supplementary material for: High-sensitivity analysis of clonal hematopoiesis reveals increased clonal complexity of potential-driver mutations in severe COVID-19 patients
Source: PLoS One. 2024 Jan 10;19(1):e0282546. doi: 10.1371/journal.pone.0282546 (PMC10781164; doi:10.1371/journal.pone.0282546)
Supplement: S1 File — (DOCX) [file pone.0282546.s001.docx]

**Supplementary information**

Supplementary information accompanying our manuscript include:

**Supplementary materials and methods**

**S1 Fig. Age distribution of individuals of our cohort.**

**S2 Fig. CH-mutations identified in the control individuals of our cohort.**

**S3 Fig. Mutational landscape of CH-mutations in our cohort.**

**S4 Fig. Survival and clinical parameters in COV-ICU patients.**

**S1 Table. List of genes included in the CHIP-UMI Panel.**

**S2 Table. Clinical parameters of COVID19 patients admitted to ICU** (submitted as separate Excel file).

**S3 Table. Detailed list of CH-mutations identified in our study** (submitted as separate Excel file).

**Supplementary References**

**Supplementary materials and methods**

**Sequencing and post-processing filtering for calling of CH variants**

We isolated peripheral blood mononuclear cells (PB-MNCs) from whole blood through density-gradient centrifugation. Genomic DNA was extracted from PB-MNCs using the DNeasy Blood and Tissue mini kit from Qiagen, following the manufacturer’s instructions.

For detection of CH variants, we designed a custom high-sensitivity sequencing assay, which uses Unique Molecular Identifiers (UMIs) for suppression of sequencing errors, called CHIP-UMI Panel. The CHIP-UMI Panel encompasses all protein-coding exons of the 80 genes most frequently mutated in 19 clonal hematopoiesis studies [1-19] (see S1 Table for the complete gene list). Sequencing library preparation was carried out with the SureSelect^XT HS^ Target Enrichment System and hybridization with the CHIP-UMI Panel’s probes, according to the manufacturer’s instructions (Agilent Technologies). Pooled libraries were sequenced on an Illumina Novaseq 6000 with 2x100 bp paired-end reads.

We validated the performance of our custom panel by sequencing reference samples which harbor known mutations at known variant allele frequencies (VAF). We used both commercially available DNA and DNA from AML patients already sequenced by whole exome sequencing (WES) in our laboratory. Using the CHIP-UMI Panel and the protocol described above, we correctly identified at the expected VAF (Linear regression: r^2^=0.96, p<0.0001) the 99% of SNVs and the 97% of indels tested. Moreover, using as ground truth the well characterized genome NA12878, for the CHIP-UMI panel, we measured a sensitivity of >100% and precision of >97%. Finally, we experimentally determined the limit of sensitivity of our assay, by serially diluting the commercially available reference samples with WT DNA, determining that our limit of sensitivity equals a VAF of 0.5% (manuscript in preparation).

Analysis of the sequencing reads was performed using the Alissa software from Agilent Technologies and aligning to the reference human genome (GRCh38). In particular, variant calling was performed with Alissa Align & Call and variant annotation with Alissa Interpret. We filtered for variants found in exonic regions of the genome, affecting the coding sequence: frameshifts and non-frameshift Indels and non-synonymous, stop-gain and stop/start loss SNVs. In order to filter for germline polymorphisms, we removed any variant reported in any population database (ESP6500, ExAC, gnomAD, 1000 Genomes Phase 3, dbSNP) with a frequency >0.005 by Alissa Interpret. We did not impose any filter on the variant allele frequency.

In order to remove further putative germline polymorphisms and potentially false-positive variants introduced by sequencing artifacts, we applied a series of post-processing filters. In particular, we took advantage of a cohort of 130 blood samples obtained from individuals enrolled in different studies designed in IEO for the analysis of clonal hematopoiesis. This cohort includes both the samples of this study and cancer patients both previous and post-chemotherapeutic treatments. All blood samples have been already sequenced with the CHIP-UMI panel and the same methodology described for the present study. All variants found mutated in ≥ 3 individuals of this cohort were included in a dataset called Likely-FalsePositive dataset. We crossed the list of CH mutations identified in the present study with the variants included in the Likely-FalsePositive dataset and removed from our final list all variants in common. Finally, variants with VAF>40%, which passed our stringent filtering strategy, very likely represent polymorphisms within the population. We retained them only if defined as potential CH drivers, according to the definition described in the paragraph entitled *Definition of potential driver CH-mutations (CH-PD)* in the Materials and methods section of the main manuscript.

**Datasets of mutations**

In order to annotate the CH variants identified by our analysis and to avoid the risk of eliminating meaningful variants for CH, i.e. variants that are frequently mutated in CH and/or myeloid neoplasms that are possibly enriched in our studies, we created two further datasets: i) the ***CH-dataset***, a dataset collecting all variants found mutated in CH, by literature mining of the studies that describe CH genomic analysis (last searched in July 2022); ii) the ***Myeloid-dataset***, a dataset of mutations in myeloid diseases, collecting all variants listed for myeloid neoplasms in cBioPortal (<https://www.cbioportal.org/>, last interrogation performed in July 2022) from the following 8 different studies, which collectively gather data coming from sequencing of 9,511 patients affected by a myeloid disease:

• Acute myeloid leukemia or myelodysplastic syndromes (WashU, 2016) [20]

• Acute Myeloid Leukemia (OHSU, Nature 2018) [21]

• Acute Myeloid Leukemia (TCGA, PanCancer Atlas) [22]

• Pediatric Acute Myeloid Leukemia (TARGET, 2018) [23]

• Myelodysplasia (UTokyo, Nature 2011) [24]

• Myelodysplastic (MSKCC, 2020) [21, 25, 26]

- Myelodysplastic syndromes (MDS IWG, IPSSM, NEJM Evidence 2022) [27]

• Myeloproliferative Neoplasms (CIMR, NEJM 2013) [28]

All variants discarded from our final list of CH mutations were further crossed with the variants included in the CH-dataset and the Myeloid-dataset. All CH mutations in common with the mutations listed in any of the two datasets were reincluded in our final list of CH variants.

**S1 Fig. Age distribution of individuals of our cohort.** COV-ICU, patients affected by COVID-19 and admitted to intensive care unit; asymPOS, pauci- or asymptomatic individuals positive to SARS-CoV-2 infection by nasopharyngeal swab and/or by detection of circulating IgGs against SARS-CoV-2; NEG, healthy individuals negative to SARS-CoV-2. The horizontal line indicates the median and the whiskers the 95% confidence interval. Statistical significance was calculated using Mann–Whitney U test: ***, p<0.01; *, p=0.016.

**S2 Figure. CH-mutations identified in the control individuals of our cohort.** A) Number of CH mutations identified in each individual. B) Variant allele frequency (VAF) of the CH mutations identified. C) Mutational landscape of CH mutations. The horizontal line indicates the median and the whiskers the 95% confidence interval. Statistical significance was calculated using Mann–Whitney U test. asymPOS, pauci- or asymptomatic individuals positive to SARS-CoV-2 infection by nasopharyngeal swab and/or by detection of circulating IgGs against SARS-CoV-2; NEG, healthy individuals negative to SARS-CoV-2 infection.

**S3 Fig. Mutational landscape of CH mutations in our cohort.** COV-ICU, patients with severe COVID-19; Controls, control group.

**S4 Fig. Survival and clinical parameters in COV-ICU patients.** Patients with severe COVID-19 are divided in patients harboring potential driver CH mutations (Driver +) and patients without potential driver CH mutations (Driver -). A) Survival analysis in COV-ICU patients. The Mantel–Cox log-rank test was used to compare survival rates. B) Clinical parameters in COV-ICU patients. The horizontal line indicates the median and the whiskers the 95% confidence interval. Statistical significance was calculated using Mann–Whitney U test.

**S1 Table. List of genes included in the CHIP-UMI Panel.**

| *ABL1* | *BRCC3* | *ETV6* | *IDH1* | *MYD88* | *PPM1D* | *SETDB1* | *SUZ12* |
| --- | --- | --- | --- | --- | --- | --- | --- |
| *ASXL1* | *CALR* | *EZH2* | *IDH2* | *NF1* | *PRDM1* | *SF1* | *TENT5C (FAM46C)* |
| *ASXL2* | *CARD11* | *FBXW7* | *IKZF1* | *NOTCH1* | *PTPN11* | *SF3B1* | *TET1* |
| *ATM* | *CBL* | *FLT1* | *JAK2* | *NOTCH2* | *RAD21* | *SH2B3* | *TET2* |
| *ATRX* | *CBLB* | *FLT3* | *JAK3* | *NOTCH3* | *RHEB* | *SMAD4* | *TNFAIP3* |
| *AXL* | *CEBPA* | *FOXP1* | *KDM6A* | *NPM1* | *RICTOR* | *SMC1A* | *TNFRSF14* |
| *BCOR* | *CREBBP* | *GATA2* | *KIT* | *NRAS* | *RIT1* | *SMC3* | *TP53* |
| *BCORL1* | *CUX1* | *GNAS* | *KMT2D (MLL2)* | *PAX5* | *RUNX1* | *SRSF2* | *U2AF1* |
| *BIRC3* | *DNMT3A* | *GNB1* | *KRAS* | *PHF6* | *SETBP1* | *STAG2* | *WT1* |
| *BRAF* | *EP300* | *HIST1H1C* | *MPL* | *PIK3CA* | *SETD2* | *STAT3* | *ZRSR2* |

**Supplementary references**

1. Busque L et al., "Recurrent somatic TET2 mutations in normal elderly individuals with clonal hematopoiesis” Nat. Genet. 2012; 44: 1179-1181
2. Shlush LI et al., "HALT Pan-Leukemia Gene Panel Consortium. Identification of pre-leukemic hematopoietic stem cells in acute leukemia." Nature 2014; 506:328-333
3. Xie M. et al., "Age-related mutations associated with clonal hematopoietic expansion and malignancies" Nat. Med. 2014; 20: 1472-1478
4. Coombs CC et al., "Therapy related Clonal Hematopoiesis in Patients with non-hematologic cancer is common and associated with adverse clinical outcomes" Cell Stem Cell 2017; 21: 374-382.e4
5. Genovese G et al., "Clonal hematopoiesis and blood cancer risk inferred from blood DNA sequence" NEJM 2014; 371:2477-2487
6. Jaiswal S et al., "Age-related clonal hematopoiesis associated with adverse outcome" NEJM 2014; 371: 2488-2498
7. Young AL et al., "Clonal haematopoiesis harbouring AML-associated mutations is ubiquitous in healthy adults" Nat. Commun. 2016; 7:12484
8. Zink F et al., "Clonal hematopoiesis, with and without candidate driver mutations, is common in the elderly” Blood 2017; 130: 742-752
9. Buscarlet M et al., "DNMT3A and TET2 dominate clonal hematopoiesis and demonstrate benign phenotypes and different genetic predisposition" Blood 2017; 130: 753-762
10. Gibson CJ et al., "Clonal Hematopoiesis associated with adverse outcomes after autologous stem-cell transplantation for lymphoma" J Clin Oncol 2017; 35: 1598-1605
11. Gillis NK et al., "Clonal hematopoiesis and therapy-related myeloid malignancies in the elderly patients: a proof-of-concept, case-control study" Lancet Oncol. 2017; 18: 112-121
12. McKerrell T et al., "Leukemia-associated somatic mutations drive distinct patterns of age-related clonal hemopoiesis" Cell Rep. 2015; 10: 1239-1245
13. Takahashi K et al., "Preleukaemic clonal hemopoiesis and risk of therapy-related myeloid neoplasms: a case-control study" Lancet Oncol 2017; 18: 100-111
14. Thol F et al., "Acute Myeloid Leukemia derived from lympho-myeloid clonal hematopoiesis" Leukemia 2017; 31: 1286-1295
15. Acuna-Hidalgo R et al., "Ultra-sensitive Sequencing Identifies High Prevalence of Clonal Hematopoiesis-Associated Mutations throughout Adult Life" The American Journal of human genetics 2017; 101:50-64
16. Arends CM et al., "Hematopoietic lineage distribution and evolutionary dynamics of clonal hematopoiesis" Leukemia 2018; 32:1908-1919
17. Coombs CC et al., "Identification of clonal hematopoiesis mutations in solid tumor patients undergoing unpaired next-generation sequencing assays" Clinical Cancer Research 2018; 24:5918-5924
18. Desai P et al., "Somatic mutations precede acute myeloid leukemia years before diagnosis" Nature Medicine 2018; 24: 1015-1023
19. Abelson S et al., "Prediction of acute myeloid leukemia risk in healthy individuals" Nature 2018; 559:400–404
20. Welch JS et al., “TP53 and Decitabine in Acute Myeloid Leukemia and Myelodysplastic Syndromes” N Engl J Med 2016; 375(21):2023-2036
21. Tyner JW et al., “Functional genomic landscape of acute myeloid leukaemia” Nature 2018; 562(7728):526-531
22. PanCancer Atlas, Cell 2018
23. Data generated by the Therapeutically Applicable Research to Generate Effective Treatments (https://ocg.cancer.gov/programs/target) initiative.
24. Yoshida K et al., “Frequent pathway mutations of splicing machinery in myelodysplasia” Nature 2011, 478(7367):64-9
25. Papaemmanuil E et al. “Genomic Classification and Prognosis in Acute Myeloid Leukemia” N Engl J Med. 2016; 374(23):2209-2221
26. Papaemmanuil E et al. “Clinical and biological implications of driver mutations in myelodysplastic syndromes” Blood 2013; 122(22):3616-27
27. Bernard et al. NEJM Evidence 2022 (International Working Group for the prognosis of Myelodysplastic Syndromes. Derivation of the Molecular International Prognosis Scoring System for MDS. Comprehensive profiling of 3,323 treatment-naive MDS samples. Data generated by the Papaemmanuil Lab 2022.)
28. Nangalia J et al., “Somatic CALR mutations in myeloproliferative neoplasms with nonmutated JAK2” N Engl J Med 2013; 369(25):2391-2405
