# Supplementary material for: High-sensitivity analysis of clonal hematopoiesis reveals increased clonal complexity of potential-driver mutations in severe COVID-19 patients
Source: PLoS One. 2024 Jan 10;19(1):e0282546. doi: 10.1371/journal.pone.0282546 (PMC10781164; doi:10.1371/journal.pone.0282546)
Supplement: Attachment — Submitted filename: Response to Reviewers_PLOSone_Final.docx [file pone.0282546.s004.docx]

*We thank the Reviewers for their valued comments and for highlighting some issues, we acknowledge that they helped us improving the quality of our manuscript.*

*In the following pages are our point-by-point responses (line # according to the revised manuscript without track changes).*

**Reviewer #1:** Doctors Chiara Ronchini, Pier Giuseppe Pelicci, and colleagues present a study of the prevalence of clonal hematopoiesis (CH)(down to a reported variant allele detection threshold of 0.5%) in 24 patients admitted to the ICU for COVID-19 (COV-ICU), and in 19 controls that include healthy subjects and those with asymptomatic, documented SARS-CoV2 infection. Major findings included the high prevalence of CH in both groups, mainly with VAF <2%, as expected by the error-corrected sequencing approach and the few previous studies that have found CH to be common in adults at VAF less than the CHIP threshold of 2%. When considering CH variants more strongly associated with driving clonal expansion and/or myeloid malignancy (CH-PD or "hazardous mutations"), the authors found a significantly higher prevalence in COV-ICU patients, as compared to controls, along with increased features of clonal complexity. Finally, the authors found no significant impact of the presence of CH-PD on clinical outcomes of ICU patients, including the probability of survival, or blood counts, clinical indicators of hemostasis or oxygen measures.

There have been several publications relating to the prevalence and clinical impact of CH in COVID-19 and the authors acknowledge these. The novelty lies mostly in the application of error-suppressed sequencing and the analysis of CH clones at lower mean VAF than previous published studies.

The major limitation, which the authors also acknowledge, is that the number of participants in their cohort is small and that this limits the ability to draw definitive conclusions. Nevertheless, their observations call for larger studies to examine (or re-examine) the potential impact of more ubiquitous, lower-VAF level CH, and prospective analysis of longer-term outcomes of COVID patients who survive ICU admission, with and without detectable CH.

These additional comments are meant to improve the quality of the manuscript:

1. It seems that the custom, error-corrected sequencing approach ("CHIP-UMI") is new and its application has not been previously peer-reviewed or published before. Otherwise, please clarify. If so, careful consideration should be given to method validation. From experience, "error-correction" is a misnomer and "error-suppression" is probably a more suitable description for such methods. The authors do present in the Supplemental Materials and Methods a limited and descriptive summary of the "validated...performance" of the custom panel. Why not show some of this data in the supplement? My concern is mainly with artifacts and "Likely-FalsePositive" findings that remain even after error suppression and filtering. Their list of CH variants, while appropriately labelled with a dedicated "Driver CH mutations" column, and colour codes to reflect levels of evidence, may still contain artifacts/errors. For example, in this small sample set the following variants appear twice: ASXL2 c.3900C>G, FBXW7 c.29T>G, KMT2D c.13040A>C, SF3B1 c74T>G. For the most part, these do not appear to be common CH or cancer-associated variants. Also, the ATRX c.5424T>G variant suspiciously appears 3 times, and while listed as "yes" for "Driver CH mutation", this reviewer cannot find reference to this ATRX variant in the CH literature. Finally, as the authors may be aware, the ASXL1 c.1934dupG (p.G646Wfs*12) variant can be contentious, and has been reported as both an artifact and true somatic variant, with the likelihood of the latter increasing with higher VAF (see PubMedID: PMID: 36652671). In this manuscript, the reported VAF is only 1.4%. Can the authors please review these variants and provide justification for their inclusion, perhaps also including an independent means (such as ddPCR) to confirm their validity, or remove them or provide further qualifications about limitations of this method?

*Response: The sequencing approach we used for this study with the CHIP-UMI panel has not been published yet. The manuscript about the validation of our approach is in preparation. We added some details on the performance of the method in the Supplementary Materials and Methods section and we changed the nomenclature to error-suppression, as suggested. We are well aware of the issue of sequencing artifacts and that is why we created a database of "Likely-FalsePositive", which we are constantly updating with new sequencing data from new individuals with the CHIP-UMI panel. Since the submission of the paper to PLOSone we increased the number of sequenced samples with the CHIP-UMI from 91 to 130.*

*We carefully reevaluated all CH variants identified in our study and, in particular, the ones highlighted by the Reviewer. We removed from our dataset ASXL2 c.3900C>G and ATRX c.5424 T>G, because with the new analysis they were found in* ≥3 *individuals and are, therefore, potential artifacts. We revisited all our data and analysis and updated all tables and figures, accordingly.*

*Concerning the ASXL1 c.1934dupG (p.G646Wfs*12) variant, we are aware of the conflicting interpretation as artifact or real somatic variant. However, based on our approach and what is reported in the literature, we believe that in our cohort it is a true somatic variant. From a technical point of view, for library preparation, we used the Agilent SureSelect kit. Enrichment of the region of interest is based on probe capture by hybridization and the kit provides the proof-reading DNA polymerase Herculase II Fusion DNA Polymerase. NGS protocols for libraries preparation with these characteristics have been shown to overcome the issue of detection of this variant as an artifact (PMID: 30222780). Moreover, we applied an error suppression protocol, obtaining for this position very good sequencing parameters: a coverage of 957 reads with >10 paired-reads supporting the alternative allele. From a biological point of view, the ASXL1 p.G646Wfs*12 true somatic variant were usually age-related (PMID: 36652671)* *and in the 94% of cases were reported in combination with other mutations with a similar VAF (median 4 somatic mutations per case, range 1±8) (PMID: 30222780). Within our entire cohort of 130 individuals, we detected this mutation exclusively in this COVID-19 patient (ICU24), who is 68 years old and harbors together with this ASXL1 variant, other 6 variants with similar VAF. In particular, two of these variants are drivers and have been reported both in CHIP and myeloid malignancies: DMT3A p.R326H and SF3B1 p.R549C.*

*All other variants highlighted by the Reviewer, within our entire cohort of 130 individuals, are still found in only two patients and, interestingly, are identified only within the COVID-19 cohort, suggesting they could be specific variants for this clinical setting. All variants are well covered (mean coverage of 830 reads and mean of 20.5 reads supporting the alternative allele). According to our analytic workflow and variant filtering, we can unbiasedly remove these variants from our dataset. Finally, none of these variants is classified as driver or hazardous mutation, therefore, they do not impact the conclusions or the implications of our study.*

2. What is the prevalence of CHIP (as defined by VAF >= 2%) in the cohorts? Without performing the analysis myself, it is not clear. The authors do state that "the vast majority of mutations...had a VAF <2%" but what about considering on a per-patient basis? I'm assuming the numbers will be too small for meaningful analysis of outcomes, but more information about traditional CHIP will allow a better comparison with previously published studies. Related to this, as compared to other studies (including Bolton et al.), the authors find here a higher proportion of TET2 variants (where Bolton and others have found more dominance of DNMT3A). Could this relate to the patient demographics, such as the number of patients with cardiovascular co-morbidity)? This may be worth discussing.

*Response: We added the results restricting our analysis to CH variants with VAF*≥*2% in the Results section (line 171 line 176). Similar to what we observed considering all CH variants, we did not score statistically significant differences between COV-ICU patients and controls. These data show that restricting our analysis to CHIP mutations has no impact on the main results and conclusions of our study.*

*Concerning the number of mutations identified in DNMT3A or TET2, in the general cohort, we scored more mutations in DNMT3A compared to TET2, as reported in most studies. If the Reviewer is referring exclusively to the COVID-19 cohort, the difference we score is of 6 mutations in TET2 vs 5 mutations in DNMT3A. We think our numbers and the differences we scored are definitively too small to be able to observe significant correlations with any clinical or demographic parameters.*

3. The lack of comparator for the 70s age group might be worth acknowledging. A recent study by Del Pozo Valero et al. found that myeloid-CH significantly increased risk of COVID-19 mortality among individuals ≥75 years, but not among those in their 60s (please see PMID: 36184726). Perhaps the lack of significance found here could be partly attributable to the relatively younger age groups that were compared. Please consider citing and discussing this study and potential limitation.

*Response: We discuss the results of this recent paper in the DISCUSSION section, line 216-225.*

4. The lack of impact of CH on clinical outcomes and parameters otherwise aligns with other studies, and the authors mention this in the manuscript. However, there may be other considerations regarding the statistically significant association of PD-CH in severe COVID-19. The authors mention "COVID-19 disease might instead select for hazardous CH-PD mutations". It might be pertinent to mention and consider the converse - that these types of PD-CH mutations could confer greater risk of severe disease, even if they’re not directly impacting prognosis while in the ICU (i.e., greater implications on host antiviral defenses rather than prolonged illness).

*Response: We added a comment in the DISCUSSION section (line 245-247)*

5. Perhaps consider mentioning earlier in the METHODS, Patient cohort section, that patient demographic information (such as age, sex etc.) is available and point the reader to the appropriate tables.

*Response: We added line 105 and 106 in the METHODS section with these pieces of information.*

6. Regarding patient demographics, the supplemental table includes the exact date of birth and date of admission. This may be too identifying. The age (in years) at presentation may be sufficient, and the authors already state that patients presented/samples were collected in April 2020. Please consider if this type of (potentially identifying) information should be removed from the supplement.

*Response: Thank you very much for* *highlighting this issue, we removed from S2 Table all potentially identifying data.*

7. Gene names should be in italics (as an example, in the Introduction, line 59; but please check elsewhere).

*Response: This has been fixed.*

**Reviewer #2:** The authors re-evaluate the link between clonal hematopoiesis and COVID-19 severity by increasing the detection sensitivity of the defining somatic mutations (threshold 0.5%) in 24 patients with severe disease, 12 asymptomatic patients and 9 non-infected donors. The tested gene panel includes 80 genes, the coverage is 1350X, COSMIC and cBioportal are used to identify driver genes with a VAF that, in more than 80% of cases, was in the 0.5-2% range. The total number of detected mutations was similar in both group. Then, potential driver mutations and clonal complexity were analyzed, and these two parameters were higher in severe patients, without significant impact on clinical and biological parameters and disease outcome in this small cohort. Altogether, this study further argue for a limited impact of clonal hematopoiesis on severe COVID-19 outcome. The manuscript is clearly written and complements a series of previous analyses with various conclusions in small cohorts. A meta analysis will be needed for solid conclusions regarding the outcome. This report is a useful contribution to prepare this analysis.
In the introduction, the authors may stick to WHO 2022 classification of CHIP (Khoury et al, Leukemia 2022): CHIP refers to somatic mutations of myeloid malignancy-associated genes detected in the blood or bone marrow at a VAF ≥ 2% (≥4% for X-linked gene mutations in males) in individuals without a diagnosed hematologic disorder and without unexplained cytopenia.

*Response: In the INTRODUCTION, line 51-55, we revisited the definition of CHIP according to the current WHO classification and cited the relevant publication, as suggested by the Reviewer.*

The WHO classification also defines clonal cytopenia of undetermined significance (CCUS) as CHIP in the presence of one or more persistent cytopenias that are otherwise unexplained by hematologic or non-hematologic conditions and that do not meet diagnostic criteria for defined myeloid neoplasms. Did some of the patients included in this trial demonstrate a cytopenia before inclusion?

*Response: None of the patients admitted to ICU had a positive clinical history for hematological disease and/or cytopenia. In the supplementary S2 Table, we report the results of the counts at admission to ICU of erythrocytes (RBC counts) and leucocytes (WBC counts). Counts are within the normal or normal-increased range for all patients.*

The introduction also focuses on the cardiovascular risk of CHIP but many other risks have now been identified, the latest being liver diseases (see B Ebert’s paper in Nature recently).

*Response: We updated the INTRODUCTION (from line 67 to line 69) and cited the above paper within the REFERENCES.*

Since they were included in 2020, do the authors have some information regarding the long-term outcome of the survivors?

*Response: At one-year post-discharge from ICU, all patients included in the study were alive. We added a column reporting this piece of information in the S2 Table.*
