# Supplementary material for: High-sensitivity analysis of clonal hematopoiesis reveals increased clonal complexity of potential-driver mutations in severe COVID-19 patients
Source: PLoS One. 2024 Jan 10;19(1):e0282546. doi: 10.1371/journal.pone.0282546 (PMC10781164; doi:10.1371/journal.pone.0282546)
Supplement: Attachment — Submitted filename: Ronchini_ResponseToReviewers_PONE-D-23-04485_R1.docx [file pone.0282546.s005.docx]

*We thank the Editor and the Reviewers for their comments. In the following pages are our point-by-point responses.*

**Journal Requirements:**

Please review your reference list to ensure that it is complete and correct. If you have cited papers that have been retracted, please include the rationale for doing so in the manuscript text, or remove these references and replace them with relevant current references. Any changes to the reference list should be mentioned in the rebuttal letter that accompanies your revised manuscript. If you need to cite a retracted article, indicate the article’s retracted status in the References list and also include a citation and full reference for the retraction notice.

*Response: we carefully reviewed our reference list and it appears complete and correct. By checking each journal website, none of the cited manuscript appears to be retracted.*

**Additional Editor Comments:**

The author should follow reviewer 1 indication and acknowledge in the final version of the manuscript the following limitations:
Small Sample Size: The study uses a relatively small sample size, which can limit the generalizability of the results. Further studies with larger patient cohorts should be undertaken to validate the findings.

*Response: We added a comment in the DISCUSSION section (line 298-301)*

Cross-Sectional Data: The study presents data captured at a single point in time, without analyzing the VAF variation over time. This cross-sectional approach might not capture the dynamic nature of clonal hematopoiesis and its implications in disease progression. Future studies should consider a longitudinal approach to track VAF variations over time to understand the role of these mutations in disease trajectory.

*Response: We added a comment in the DISCUSSION section (line 301-305)*

Age-Dependent Variation: While the study acknowledges age-dependent variations, it seems the age groups compared have different sample sizes, which might affect the statistical power of the analysis. Implementing age-matched control groups would strengthen the study.

*Response:* *One of the aims of our study was to analyze and compared samples from COV-ICU patients and controls that were collected during the same phase of the Covid-19 pandemic in the same regional area, in order to minimize the probability of infections from different viral variants, that clearly showed different microbiological characteristics and, more importantly, completely different clinical impacts on the infected individuals. Moreover, we wanted to analyze controls collected before the vaccination campaign, again, in order to avoid possible confounding effects on the hematopoietic cell system of our samples. In order to fulfill these requirements, for the controls, we took advantage of the study SOS-COV2 that included personnel working at our Institute and* *we analyzed all available samples collected from the oldest individuals of this cohort.*

Clinical Significance of CH-PD: The study identified a higher percentage of CH-PD mutations in the COV-ICU group compared to the control group; however, the clinical significance of these mutations, in terms of their impact on patient outcomes, remains unclear. The manuscript could benefit from a deeper analysis examining the clinical relevance of these findings, potentially linking it to patient outcomes or specific clinical characteristics.

*Response: In the manuscript all available clinical data, patient outcome and follow-up at one year for the COV-ICU cohort are included (S2 Table). As shown in S4 Figure entitled: “Survival and clinical parameters in COV-ICU patients”, comparing COV-ICU patients with CH-PD mutations versus patients without CH-PD mutations, we did not identify statistically significant differences. This is acknowledged in the Results section (line 201-203). We added the reference to S2 Table and the no significance of data.*

Lack of Long-Term Follow-Up: The study does not follow up on the potential development of more serious conditions like MDS/AML based on the identified mutations, missing an opportunity to explore the long-term implications of the identified CH-PDs.

*Response: The absolute risk of hematopoietic malignancy development in persons with CH is low and individuals with CH progress to malignancy at a rate of about 0.5 to 1% per year. It has been reported that 4% of persons with CH develop hematopoietic malignancies in 8 years (Jaiswal et al., NEJM 2014). In the study from Abelson et al. (Nature 2018) individuals with CH developed myeloid neoplasms with a median follow-up of 7.6 years. Moreover, quantitatively the authors found that CH-PD mutations conferred around a 2-fold increase risk of developing AML per 5% increase in clone size. Moreover, CH parameters that increase the risk of leukemia transformation are linked to the presence of: clones with VAF>10%, more than 1 mutated gene, mutations in specific driver genes and specific aminoacid changes. Considering that: i) our cohort is small, ii) the COV-ICU patients had normal hematological counts and iii) in our COV-ICU patients the majority of clones have VAF<2%, we believe that the probability of scoring the development of myeloid neoplasms in the time frame of follow-up of 3 is extremely low. Moreover, although very interesting and important, this is beyond the scope of our study, which was the assessment of CH at high sensitivity in the two cohorts.*

**Implementations that will be needed in future studies (if beyond the scope of this manuscript):**
Longitudinal Study: Implement a longitudinal study design to better assess the variations of VAF over time and potentially capture more nuanced relationships between CH, age, and disease severity.

Expanded Genetic Analysis: Consider an expanded analysis involving a greater number of genes to potentially uncover more associations between genetic variations and disease outcomes.

Multivariate Analysis: Incorporate multivariate analyses to control for potential confounding factors and to robustly examine the association between the identified mutations and disease outcomes.

*Response: We agree with the editor that all these issues are very insightful. However, they would require the design and development of new specific trials and tools that are beyond the scope of this manuscript.*

**Questions to be Addressed**
VAF Variation over Time: The authors should discuss if and how VAF variation over time (months/years)can influence the outcomes and the observed CH. This can potentially be a marker for disease progression and should be considered in future studies.

*Response: We added a comment in the DISCUSSION section (line 236-250)*

Relation to CHIP/CCUS ICUS: It would be beneficial for the authors to discuss whether the allele frequencies observed can be a mirror of CHIP/CCUS ICUS or frankly MDS/AML in some or other cases. An acknowledgment of this potential association and a suggestion to explore this in future research would enrich the discussion.

*Response: Most of the genomic studies on myeloid neoplasms are based on whole exome or large gene panels sequencing analysis and usually, consistent with normal clinical practice, use VAF threshold of 1% for hotspot mutations and of 5% for variants of unknown significance. Moreover, in frank myeloid neoplasms usually important clonal expansions has already taken place. It is, therefore, not surprising that most of the mutations in common with our study display higher VAF in dataset of myeloid neoplasms or CHIP compared to the one we scored. Nonetheless, we found similar VAF in a few patients with myeloid neoplasms and we added a comment addressing this issue in the DISCUSSION section (line 256-268).*

**Reviewer #1:** I thank the authors for considering and addressing the majority of my comments. I am satisfied with the overall changes and believe these have improved the manuscript.

I do ask the authors to check the numbering of references and to ensure this has not been disrupted. This came to my attention when following references 30-32, the criteria for CH and PD-CH variants. In the original submission, these correctly referred to studies by Bolton et al., Young et al. and Acuna-Hidalgo et al. If I am not mistaken, the numbering of references has shifted in this revised version, and now 30-32 correspond to Abelson et al. (prediction of AML risk in CH), Fabre et al. (study of CH in twins), and Bolton et al. I do not believe this is correct. This problem may be more extensive than mentioned. Please review carefully reference numbers in text versus the reference list to ensure matching. Thank you.

*Response: The numbering of references changed from the original manuscript because, as mentioned in the Rebuttal letter for the first round of revision, we added new references, according to the suggestions by the Reviewers. We carefully checked the numbering reported in the revised manuscript and it is correct. Indeed, for annotation of CH variants as potential drivers, we used the definitions and the parameters reported in Bolton et al., Abelson et al. and Fabre et al. In particular, we used as reference the Supplemental Table 2 published in Fabre et al. plus all parameters described in the other manuscripts. To the best of our knowledge, there is no a formal definition of CH-Driver mutations in Young et al. or Acuna-Hidalgo et al.*
