# Supplementary material for: High-sensitivity analysis of clonal hematopoiesis reveals increased clonal complexity of potential-driver mutations in severe COVID-19 patients
Source: PLoS One. 2024 Jan 10;19(1):e0282546. doi: 10.1371/journal.pone.0282546 (PMC10781164; doi:10.1371/journal.pone.0282546)
Supplement: Attachment — Submitted filename: Ronchini_ResponseToReviewers_PONE-D-23-04485R2.docx [file pone.0282546.s006.docx]

*In the following pages are our point-by-point responses.*

**Journal Requirements:**

Please review your reference list to ensure that it is complete and correct. If you have cited papers that have been retracted, please include the rationale for doing so in the manuscript text, or remove these references and replace them with relevant current references. Any changes to the reference list should be mentioned in the rebuttal letter that accompanies your revised manuscript. If you need to cite a retracted article, indicate the article’s retracted status in the References list and also include a citation and full reference for the retraction notice.

*Response: we double-checked the in-text citations against the updated reference list and ensured that all references are accurately represented. By checking each journal website, none of the cited manuscript appears to be retracted.*

**Additional Editor Comments:**

Overall, the authors have made efforts to address the reviewers' comments and justify any limitations or decisions made in the study, while also updating the reference list and clarifying any confusion around it. They are open about the limitations of their study and have incorporated suggestions from the reviewers into the revised manuscript where possible. They also highlight areas that could be explored in future research but which were beyond the scope of their current study. Indeed, the authors' responses in the rebuttal letter generally address the concerns raised by the reviewers. However, there are always areas for improvement, both in the manuscript and in the way the authors communicate their revisions to the reviewers and the readers. Here are some suggestions:

**Clarification on Reference Numbering:**

The response about the reference numbering was somewhat unclear. The authors should make sure that the reference numbers in the text match the updated reference list. They should explicitly confirm that they have double-checked the in-text citations against the updated reference list to ensure all references are accurately represented.

*Response: we confirm that we double-checked the in-text citations against the updated reference list and ensured that all references are accurately represented.*

**Explicit Acknowledgement of Limitations:**

While the authors have acknowledged the limitations pointed out by the reviewers in the discussion section, it may be beneficial to include a succinct summary of these limitations in the abstract or conclusions to ensure readers are immediately aware of the study's scope and constraints.

*Response: we acknowledged the limitations of our study in the abstract (line 49-53).*

**Methodological Rigor and Follow-Up:**

Although the authors mention that long-term follow-up and expanded studies are beyond the scope of the current study, they could outline a more detailed plan for future research. This might include potential study designs, collaborations, or funding opportunities they plan to seek in order to address these limitations.

*Response: A possible strategy would be to retrospectively retrieve DNA from PB that have been collected from COVID19 patients treated in ICU and, then, longitudinally at different follow-up post-discharge, in order to perform our high sensitivity CH analysis. However, we really believe this is beyond the scope of the current study.*

**Detailed Justification for Methodological Choices:**

For the controls chosen from the SOS-COV2 study, the authors should provide a more detailed rationale for why these specific samples were selected, beyond the timing of collection and the pre-vaccination status, such as demographic similarities or exposure risks that match the study group.

*Response: we added these details in the Materials and Methods section (line 110-118).*

**Statistical Considerations:**

The authors should consider discussing any additional statistical tests or models that could be applied to their data in future studies to account for confounding variables not addressed in this study, such as multivariate regression analyses or propensity score matching.

*Response: Increasing the number of patients analyzed, one could apply machine learning models to discriminate between ICU patients and Controls by integrating multivariate clinical features with our genomic features. However, we believe it is premature to suggest any additional specific type of statistical tests or models without additional available information.*

**Potential Implications and Applications:**

A deeper exploration of how the identified mutations could be monitored or used in clinical practice might add value to the discussion, even if this application is not immediately feasible.

*Response: We applied a high sensitivity NGS technology which requires extensive experimental and bioinformatic workflows, is expensive, and is not easily implementable in clinical practice. To our knowledge, up to day, the only alternative available technique with similar sensitivity is digital PCR, not used in standard clinical practice. Moreover, how to counsel people with CH or monitor them prospectively is an area of uncertainty and a quite challenging task at the moment. Indeed, there are no clinical indications or guidelines on how to manage the presence of CH mutations nor there are established interventions to try to eradicate CH clones. We believe it is really too early to include this information in our paper, especially, considering that we mainly detected low frequency mutations, which require high sensitivity techniques to be monitored.*

**Accessibility of Data and Materials:**

Ensuring that all supplementary materials and methods are easily accessible to readers and researchers for reproducibility purposes is important. If the authors have not already done so, they might consider including a statement confirming the availability of such resources or outlining how they can be accessed.

*Response: we added a Data Availability Statement (line 322-324).*
